# Exploring the Role of Masturbation as a Coping Strategy in Women

**DOI:** 10.1080/19317611.2024.2344812

**Published:** 2024-04-29

**Authors:** Fabienne S. V. Wehrli, Guy J. Bodenmann, Joëlle Clemen, Katharina Weitkamp

**Affiliations:** Clinical Psychology for Childern/Adolescents and Couples/Families, Department of Psychology, University of Zurich, Zurich, Switzerland

**Keywords:** Masturbation, psychological distress, coping, clitoral and vaginal stimulation, concurrent mixed-method design

## Abstract

**Objectives:**

Recent findings suggest that individuals tend to engage in masturbation more frequently when experiencing elevated levels of psychological stress, and there appears to be distinguishable effects on stress response based on clitoral and vaginal stimulation. In this concurrent mixed-method study, we aimed to investigate this association in more detail using a convenience sample of 370 women.

**Methods:**

Quantitative data were used to examine whether higher psychological distress was associated with higher levels of masturbation frequency depending on the mode of stimulation, while qualitative data gave further insight into this association.

**Results:**

In regression analysis, higher levels of general and subscale-specific psychological distress were significantly associated with higher clitoral, but not combined clitoral and vaginal masturbation frequency. Qualitative content analysis showed that masturbation was used as a reliable coping strategy and self-care strategy which induced positive affective states, such as happiness and relaxation. Very few women reported negative feelings associated with masturbation. Mixed-method analysis revealed that women who indicated to use of masturbation for coping or self-care or who reported negative feelings did not differ in their level of psychological distress from women who did not report using it. The positive effects of masturbation were not related to the mode of stimulation.

**Conclusions:**

Results showed the complexity of how psychological distress is related to sexual activity and point to the potential benefits of masturbation for dealing with psychological distress and for enhancing general well-being. Our results have various implications for researchers, clinicians, and society.

## Introduction

Masturbation is a very common sexual behavior among women in Western societies. In nationally representative surveys conducted in the UK (Gerressu et al., 2008), US (Herbenick et al., 2023), and Australia (Richters et al., 2006), prevalence rates of masturbating at least once in the past year have ranged from 21 to 42% among adult women. However, masturbation has long been stigmatized across societies and religions, with women being especially discouraged or even prohibited from exploring their sexuality (Rowland et al., 2020). To this day, this stigmatization continues to influence the debate about the association between women’s sexual activity and emotional well-being. For instance, masturbation behavior in women was perceived as harmful and labeled as the cause of a multitude of physical disorders and psychological distress by several health professionals (Freud, 1905; Kay, 1992). In contrast, other scholars proposed that masturbation should be conceptualized as beneficial to one’s sexual and overall well-being (Dickenson & Huebner, 2016).

The beneficial effects of masturbation on mood and the use of masturbation to relief stress have previously been reported in women. For example, physiological and emotional responses to sexual arousal, orgasm, and sexual activity are associated with stress regulation and improved mood (Alley et al., 2019; Burleson et al., 2007; Cera et al., 2021). Clitoral masturbation and the resulting orgasms may have mood-enhancing effects, whereas masturbation involving vaginal stimulation and the resulting orgasms might have a more relaxing and calming effect (Mah & Binik, 2001; Prause et al., 2016; Sayin, 2017). Thus, it may be speculated that masturbation provides a beneficial way of coping with psychological distress.

These findings challenge the narrative of women’s masturbation as the cause of mental illness and propose that masturbation should be conceptualized as beneficial to one’s overall well-being (Dickenson & Huebner, 2016). However, few studies have investigated the role of clitoral and vaginal stimulation in the context of using masturbation for stress reduction.

### Masturbation and psychological distress

Psychological distress is broadly defined as a state of emotional suffering characterized by symptoms of depression and anxiety (Mirowsky & Ross, 2002). Recent studies found a higher masturbation frequency among women with clinical and subclinical levels of psychological distress compared to non-clinical groups. When examining clinical levels of psychological distress, one study compared sexual functioning among 48 non-medicated women with moderate to severe depression (scoring between 20 and 38 on the Beck-Depression-Inventory) with an age-matched control group. Women with depressive symptoms reported more problems with sexual functioning than their matched controls, but they expressed a higher desire for masturbation than the non-depressed group. Exploratory analysis showed that the groups did not differ in desire for sexual activity with a partner (Frohlich & Meston, 2002). Likewise, another cross-sectional study with 914 US women found that masturbation frequency was significantly higher in women with a lifetime history of major depression disorder compared with women with no such history. Again, groups did not differ in their frequency of partnered activity (Cyranowski et al., 2004). Similar results were reported among women with subclinical levels of psychological distress. For example, a recent study with 2215 women (most of them having current sexual partners) in the USA found that a higher frequency of masturbation was related to higher levels of general anxiety and depressive symptoms (Rowland et al., 2020). Lastly, the frequency of masturbation correlated significantly with trait anxiety and depression in a sample of 96 women diagnosed with polycystic ovary syndrome in Poland (Glowinska et al., 2020). Thus, some scholars argue that masturbation increases negative feelings, which is why it should be considered problematic and harmful (Brody & Nicholson, 2013; Castellini et al., 2016). An alternative explanation for these findings is that masturbation might function as a reliable form of self-soothing behavior during times of stress (Brotto et al., 2010; Frohlich & Meston, 2002; Maatouk et al., 2022; Rowland et al., 2020). While it has previously been well established that men use masturbation as a way to cope with stress (Engel et al., 2019; Leonard, 2010; Miner et al., 2019; Wilson et al., 2022), research about this phenomenon in women is scarce. Lately, this has received more consideration in both literature and public discourse (e.g., Burri & Carvalheira, 2019), and this study aims to shed more light on this question as well.

### Masturbation as a coping strategy

Masturbation is a safe, free, and accessible form of sexual activity that does not rely on the availability of or emotional interaction with another person (Frohlich & Meston, 2002; Scimeca et al., 2013). As a solo activity, it is less likely to evoke concerns about performance or evaluation that may arise during sexual activities with a partner. Instead, women can fully focus on themselves. This might be especially relevant for women since previous research suggests significant gendered inequalities around sexuality (Ford et al., 2023). As such, sexual interactions are reported to generally be less pleasurable and accompanied by greater costs for heterosexual women compared to heterosexual men (Laan et al., 2021). Additionally, there is a well-documented orgasm-gap in sexual interactions between heterosexual men and women (Frederick et al., 2018). Even though the evidence is conflicting, some studies have shown a complementary relationship between masturbation and partnered sexual frequency, indicating that masturbation appears to be distinct from partnered sexual activities in women (Fischer & Træen, 2022; Regnerus et al., 2017). One study showed that women tend to reach orgasms more easily during masturbation as compared with partnered sexual activity (Rowland et al., 2018). Therefore, the association between psychological distress and masturbation may be different from the one between psychological distress and partnered sexual activities (Bodenmann et al., 2010).

The concept of masturbation as a self-soothing behavior has been explored in numerous developmental studies investigating masturbation during infancy, childhood, and adolescence. Findings suggest that children tend to engage in masturbation more frequently during times of stress, for example during the weaning process, the arrival of a new sibling, or parental separation (Friedrich et al., 1998; Schoentjes et al., 1999; Unal, 2000). There are various psychological reasons to suggest that masturbation is related to stress reduction and mental well-being. Masturbation (and especially orgasm) can have a positive impact on women’s affective states. Some studies have reported that masturbation leads to feelings of happiness, relaxation, autonomy, control, and contentment, eliciting positive affective states (Goldey et al., 2018; Meiller & Hargons, 2019). Masturbation has also been repeatedly mentioned as beneficial to mood regulation and stress reduction (Burri & Carvalheira, 2019; Csako et al., 2022; de Lima et al., 2022; Graham et al., 2004). Studies have shown that women use masturbation to reduce and overcome negative affective states, such as anxiety and depression (Csako et al., 2022; Gouvernet et al., 2021; Graham et al., 2004). Other motivations to engage in masturbation include the desire to deal with physical pain (Leistner et al., 2023; Meiller & Hargons, 2019) or to facilitate falling asleep (Carvalheira & Leal, 2013). Taken together, both the developmental data and the many studies on adult’s reasons for masturbation suggest that masturbation might function as a coping strategy during times of stress. Interestingly, studies have also suggested that the effects of mental health and stress reduction may vary depending on whether clitoral or vaginal stimulation is involved.

### The role of clitoral and vaginal stimulation in stress regulation

Women’s masturbation typically only involves stimulation of the clitoris (Adaikan, 2017; Dekker & Schmidt, 2003; King et al., 2011; Regnerus et al., 2017), or includes both clitoral and vaginal stimulation simultaneously (Butler, 1976; Clifford, 1978; Prause et al., 2016). Research exploring the potential beneficial effects of clitoral and vaginal stimulation is still limited. Rowland et al. (2020) found that women who exclusively used clitoral stimulation during masturbation were more likely to report stress relief and relaxation as the primary motivations. Moreover, research indicates clear differences in physiological and psychological effects resulting from clitoral and vaginal stimulation. Physiologically, research has shown that clitoral and vaginal stimulation result in different activation patterns in the homuncular map in the somatosensory cortex. Clitoral stimulation primarily leads to activation of the pudendal nerve, while proximal-vaginal and cervical stimulation mostly results in activation of the pelvic, hypogastric, and vagal nerves (Jannini et al., 2018; Komisaruk et al., 2004; Whipple & Komisaruk, 2002). Vagal nerves are known to influence the parasympathetic system and heart rate during stress (Jannini et al., 2012; Komisaruk et al., 2004). These different physiological effects might translate into different psychological effects. For example, qualitative self-reports have described orgasms achieved through clitoral stimulation as electric, short-lasting, joyful, and exciting, while orgasms resulting from vaginal stimulation have been described as stronger, longer-lasting and more relaxing (Butler, 1976; Clifford, 1978; Jannini et al., 2012; Mah & Binik, 2005; Palmer, 2014; Sayin, 2017). Furthermore, studies indicate that clitoral stimulation is often considered the fastest, easiest, and most reliable means of sexual arousal (Jannini et al., 2012; King et al., 2011; Sayin, 2017), which makes it likely to be utilized for stress relief. Therefore, rather than being the cause of psychological distress, it can be hypothesized that clitoral masturbation might have mood-enhancing effects, while masturbation including vaginal stimulation might have a more relaxing and calming effect.

The empirical evidence suggests that frequent masturbation might serve as a reliable form of stress relief for women experiencing higher levels of psychological distress. However, some researchers question the beneficial effects of masturbation by pointing to the association between mental health symptoms and masturbatory behavior. To date, no study has investigated the association between masturbation and psychological distress while also exploring the reasons for engaging in masturbation within the same sample to shed light on these potentially contradictory findings. Furthermore, the existing evidence regarding the link between the mode of stimulation and psychological distress remains inconclusive and warrants further investigation. It is crucial to differentiate between masturbation only using clitoral and additionally incorporating vaginal stimulation in this context. Understanding these associations in more detail can help inform future efforts to promote sexual and mental health in women and give information on how different symptoms of psychological distress are related to masturbation behavior.

### The current study

Up until now, to the best of our knowledge, no studies have investigated the use of masturbation as a self-care strategy to attain stress reduction in women and the role of clitoral and vaginal stimulation in this context. The present study sought to provide further information on the matter by explaining quantitative survey results with qualitative open-ended questions in a convergent mixed-method design. Our quantitative research question aims at understanding the link between psychological distress and masturbation frequency.

- We hypothesized that (1) higher psychological distress is associated with higher masturbation frequency in women. We expected this association for two modes of stimulation (clitoral; combined clitoral and vaginal).
- Using qualitative data, we further explored (2) in what ways expressed feelings about masturbation in women’s self-reports help understand the relationship between psychological distress and masturbation frequency.

Finally, we integrated the quantitative and qualitative results within a mixed-method approach. For this purpose, four mixed-method research questions were formulated after analyzing both quantitative and qualitative data.

- We expected that (3) psychological distress is higher in women who report using masturbation as a coping strategy or for their personal well-being in qualitative self-reports compared to women who do not indicate these categories.
- We also checked for the possibility that (4) negative effects of masturbation are associated with psychological distress.
- Furthermore, we expected that in women’s qualitative self-reports, (5) women using clitoral stimulation are more likely to indicate uplifting and joyful emotions after masturbation than women incorporating additional vaginal stimulation.
- (6) Women incorporating additional vaginal stimulation during masturbation are more likely to report relaxing and calming feelings after masturbation than women using clitoral stimulation.

## Methods

### Participants

Participants were a convenience sample of 370 women (range 18–56 years old, *M*** **= 26.04, *SD*** **=** **5.74) who reported engaging in masturbation within the past three months. All sample characteristics are listed in Table 1. The sample primarily consisted of young, heterosexual (85%), non-religious (55%) women who were in a close relationship (64%) and never married (83%). Close to half of the participants held a university degree.

**Table 1.** Sociodemographic variables.

Variable	Participants	
	*n*	%
Education		
Apprenticeship	18	5
High school/secondary school/BMS	126	34
Higher school of education	48	13
University/ETH/PH	175	47
Other	3	1
Nationality		
Switzerland	317	86
Germany	16	4
Austria	12	3
Other	25	7
Religion		
Christianity	186	50
Judaism	1	0
Islam	6	2
Buddhism	0	0
No religion	173	47
Other	4	1
Practicing religion		
Not religious	203	55
A little religious	102	28
Medium religious	48	13
Very religious	12	3
Highly religious	5	1
Civil status		
Never been married	306	83
Married	32	9
Registered partnership/concubinage	19	5
Separated/divorced	6	2
Other	7	2
Sexual orientation		
Heterosexual	315	85
Lesbian	6	2
Bisexual	34	9
Pansexual	10	3
Other	5	1
Relationship status		
Close relationship	237	64
Single	112	30
Other	21	6
Cohabitation		
Yes	107	29
No	151	71
Children		
Yes	32	9
No	338	91
Sexual assault		
Yes	80	22
No	280	76
Don’t want to answer	10	3
Hormonal contraception		
Yes	155	42
No	256	58
Breastfeeding		
Yes	5	1
No	365	99

### Procedure

To test the hypotheses, we implemented a concurrent (convergent) mixed-method design. Mixed methods research has gained momentum in the past years (Anguera et al., 2018; Cameron, 2011) and is defined as the combined collection, analysis, and interpretation of quantitative and qualitative data for the same study purpose (Anguera et al., 2018). This allows for a holistic understanding of the data by combining approaches and strengths of either methodology (Turner et al., 2017). The concurrent mixed-method design uses simultaneously collected quantitative and qualitative data from the same study population (Creswell et al., 2003). The goal is to identify converging evidence to enhance the validity of the conclusions drawn from different methods (Creswell et al., 2003; Kajamaa et al., 2020). We integrated the qualitative and quantitative paradigms by following the stance of dialectical pluralism (Johnson, 2017) meaning we relied on multiple methods, paying careful and deep attention to multiple sides of the research processes, striving for integration of potentially opposing results through respectful dialog of qualitative and quantitative research strands.

We used cross-sectional data that were collected as part of a larger study on women’s orgasms in Switzerland. The data was not originally collected for the purpose of this study. The study was conducted following the Ethical Principles of Psychologists, the American Psychological Association’s (APA) Code of Conduct, and the Swiss Psychology Association and was approved by the Institutional Ethical Review Board of the University of Zurich on December 16, 2020. Data collection was started and completed in January 2021 via an anonymous online survey (Questback EFS Fall 2020) which was advertised on different social media channels (Instagram and Facebook) and distributed through mailing lists of the Universities of Zurich and Bern, respectively. Additionally, we asked interested individuals to forward the link to their personal networks. Individuals were eligible for the study if they were at least 18 years of age, fluent in German, identifying as cisgender women, and sexually active in the past three months (masturbation and/or sexual intercourse). Interested individuals confirmed their eligibility and provided informed consent before starting the survey, which took ∼35 min to complete. We gave participants the option to provide their e-mail addresses separately from participant responses to be entered into a prize draw (including a book and a 20 CHF voucher for a bookshop) or to get university credits. Participants completed items related to their sociodemographic background and medical history, study-specific questions on masturbation behavior as well as questionnaires on their sexuality (e.g., sexual function (Female Sexual Function Index; FSFI-d); Berner et al., 2004), and psychological distress. Participants also answered qualitative open-format questions. Of 844 participants who began the survey, 514 women completed it (62%). Due to the recruitment methods (snowballing, social media recruitment), calculating a response rate was not possible. One person was excluded for not fitting the inclusion criteria (male sex as indicated by the individual in the open-ended questions). For the current analyses, 15 participants (3%) were excluded because they never masturbated in their life, and another 53 (11%) participants were excluded because they had not masturbated within the last three months. Furthermore, we excluded participants under current psychiatric medication (*n*** **=** **22, 5%) to ensure that sexual desire was not pharmacologically affected (e.g., by antidepressant medication). This resulted in a sample size of 423 women. We excluded women who relied solely on vaginal stimulation because there was insufficient statistical power to establish a separate group for this mode of stimulation (*n*** **=** **6, 1%). Additionally, we excluded women who reported using anal stimulation (*n*** **=** **21, 5%) or other forms of stimulation (*n*** **=** **3, 1%) in a representative month. This decision was made because otherwise, we would not be able to distinctly attribute the effects of masturbation in the qualitative data to the specific mode of stimulation (clitoral *vs.* combined clitoral and vaginal) in these participants. Lastly, we excluded women that indicated to have used clitoral stimulation next to combined clitoral and vaginal stimulation for the same reason (*n*** **=** **47, 11%). This process resulted in a final sample size of *N*** **=** **370 women for regression analysis (*n*** **=** **256 engaging in clitoral stimulation and *n*** **=** **114 engaging in combined clitoral and vaginal stimulation). To check for the stability of the results, we reran the analysis adding the above-mentioned excluded subgroups to the sample. Overall, this did not influence the results, see Open Science Framework (OSF) (https://osf.io/cgys2/?view_only=a740e72afb794f148b4a1db8489a6112).

### Measures

The variables presented here were part of a larger study on female sexuality in Switzerland, which incorporated additional measures. The variables masturbation frequency, psychological distress, and one qualitative question used for the analyses in this study are listed here. For further information on other variables and previously published work with the sample, please refer to the OSF (https://osf.io/cgys2/?view_only=a740e72afb794f148b4a1db8489a6112).

#### Sociodemographic information and covariates

Participants answered several questions regarding age, education, nationality, religiosity, civil and relationship status, sexual orientation, cohabitation, having children, sexual assault in the past, hormonal contraception use, and breastfeeding.

#### Masturbation frequency

At the beginning of the questionnaire, we asked participants to indicate whether they had ever masturbated or not. In case they reported having masturbated, we provided participants with a graphical image of female reproductive organs to ensure a common knowledge base and then asked them to indicate which mode of stimulation they mainly used during masturbation in one representative month during the past three months (available from the OSF; https://osf.io/cgys2/?view_only=a740e72afb794f148b4a1db8489a6112). Participants could choose whether they focused on clitoral stimulation, vaginal stimulation, clitoral and vaginal stimulation, anal stimulation, or other stimulation. Multiple answers were possible. Participants then indicated masturbation frequency for the chosen mode of stimulation (clitoral, vaginal, and combined clitoral and vaginal stimulation). Masturbation frequency was defined as recalled masturbation days during the chosen representative month.

#### Psychological distress

Psychological distress was measured using the Symptom-Checklist-27 (SCL-27; Hardt et al., 2011). Participants responded to the 27 items on a Likert scale with anchors from 1 (not at all) to 5 (very strongly) asking about psychological distress in the past seven days (Hardt et al., 2014). High temporal stability of the measure allows interpretation of the scores on the trait level (Hardt et al., 2011; Klaghofer & Brähler, 2002). The SCL-27 contains six subscales *depressive*, *dysthymic*, *vegetative*, *agoraphobic*, *sociophobic symptoms*, and *symptoms of mistrust* with four to six items per subscale (Hardt et al., 2014). Scale scores are calculated from the mean value of the corresponding items while missing item values are replaced by scale mean scores (Hardt et al., 2014). Furthermore, a global severity index (GSI-27) was calculated in the same manner (Hardt et al., 2014). Psychometric properties were validated in a German sample (Hardt et al., 2014) and reliability analysis in this study yielded Cronbach’s alphas of *α* > .68 for subscales and a Cronbach’s alpha of *α* = .90 for the GSI-27.

#### Feelings about masturbation

To gain insight into women’s personal feelings associated with masturbation, an open-ended question was posed at the end of the survey. We specifically asked women: “What does masturbation make you feel like?” We did not specifically ask women about their use of masturbation as a coping strategy to reduce confirmation bias, as suggested in qualitative research (Powell et al., 2012; Roulston & Shelton, 2015). Women were asked to take their time in answering the question and were assured that there were no right or wrong answers.

### Data analysis

#### Quantitative data analysis

Power analysis for regression analysis revealed that at least 139 participants per mode of stimulation would be needed to ensure appropriate power for a medium effect size and a significance level of 0.05. The association between psychological distress and masturbation frequency was investigated with separate regression analyses for each mode of stimulation (clitoral/combined clitoral and vaginal). We included covariates in the association between psychological distress and masturbation frequency. After consulting existing literature, the variables age (e.g., Robbins et al., 2011), education (e.g., Gerressu et al., 2008), relationship status (e.g., Das, 2007; Fischer et al., 2022), cohabitation (e.g., Brody et al., 2000), sexual orientation (e.g., Bowman, 2014), hormonal contraception use (e.g., Botzet et al., 2021), practicing religion (e.g., Baćak & Štulhofer, 2011), sexual assault (e.g., Csako et al., 2022), and having children (e.g., Dewitte et al., 2017) were considered as covariates. We ran the regression analysis including covariates that were significantly associated with masturbation frequency, as determined by correlation analysis, Kruskal-Wallis-tests, and *t*-tests where applicable. The included covariates were dummy-coded. Preliminary analysis with Shapiro-Wilk tests showed a violation of the normal distribution of residuals. Therefore, we additionally computed bootstrap regression analysis using 95% confidence intervals around the estimates with 5000 bootstrap samples (Preacher & Hayes, 2004). All statistical analyses were executed using R (version 2022.07.2). The data and code itself—including further analyses (e.g., sample including women who did not masturbate in the past)—are available from the OSF (https://osf.io/cgys2/?view_only=a740e72afb794f148b4a1db8489a6112).

#### Qualitative data analysis

The qualitative data were analyzed by two trained raters. Working with categories (codes), Qualitative Content Analysis (QCA; Kuckartz, 2019) was performed on the open-ended questions. Categories were developed either concept-driven (deductive) from previous research presented above (e.g., using masturbation as a coping mechanism as proposed by Burri & Carvalheira, 2019) or data-driven (inductive) when the content in the data called for the formation of additional categories (Kuckartz, 2019). The creation of categories and coding of data took place in several cycles by both raters. The last author, who is versed in qualitative data analyses, audited the process. The final coding frame consisted of the deductively formed categories that were complemented by the data-driven categories. The complete material was independently double-coded by the two raters using the final coding frame, which resulted in high inter-rater reliability for all codes (87–98%). The remaining differences in code allocation were discussed until a consensus was reached and subsequently assigned to the final category. Because of the large quantity of material, we focused on those statements in the material relevant to the specific research questions of this study. Typical answers from women were used to demonstrate the core concept of a category (Kuckartz, 2019).

#### Mixed-method analysis

To integrate quantitative and qualitative data, we used dichotomization, which is an emerging strategy of more complex mixed methods research (Creswell et al., 2003; Driscoll et al., 2007; Nzabonimpa, 2018; Srnka & Koeszegi, 2007). Dichotomization means that numerical values are assigned to each participant in terms of the presence (1) or absence (0) of a qualitative code (Driscoll et al., 2007). Thus quantified as dichotomous codes, qualitative data can be statistically integrated and analyzed with quantitative data, which allows for deeper insights into the data (Driscoll et al., 2007).

In this study, we dichotomized four categories that were established from QCA and that were relevant to the research question. We performed one-sided two sample *t*-tests to determine whether women that received a code for the category *using masturbation as a coping strategy* or *using masturbation for well-being and self-care* differed in the level of psychological distress from women that did not receive a code for the same category. The same was performed for group differences between women who received a code for the category *negative effects of masturbation* and women who did not. We used a significance level of *p* < .001 to correct for multiple testing. Furthermore, we used four Chi-Squared tests to examine if the mode of stimulation (clitoral/combined clitoral and vaginal) was related to the categories *evoking joyful and uplifting emotions* or *evoking calming and relaxing emotions*. We applied a significance level of *p* < .05.

## Results

### Quantitative results

#### Descriptive statistics

Of the 370 women included in this study, descriptively, more women used clitoral stimulation (*n*** **=** **256, 62%) than combined clitoral and vaginal stimulation (*n*** **=** **114, 27%). Table 2 shows masturbation frequencies, psychological distress, and sexual function for the mode of stimulation. Welch-two sample *t*-tests (two-sided) showed that masturbation frequency in a representative month was significantly higher in women using clitoral stimulation as compared to women using combined clitoral and vaginal stimulation (*t*_(282)_ = 3.10, *p* = .002, Hedges’ *g*** **=** **0.30). There were no significant differences in levels of psychological distress or sexual function between the two groups.

**Table 2.** Masturbation frequency, psychological distress, and sexual function depending on mode of stimulation.

Variable	Masturbation frequency				Psychological distress				Sexual function			
	*M*	*SD*	Min.	Max.	*M*	*SD*	Min.	Max.	*M*	*SD*	Min.	Max.
Clitoral stimulation (*n* = 256, 62%)	9.19	7.19	1	30	0.56	0.41	0.00	2.26	27.66	5.36	3.80	35.40
Combined clitoral and vaginal stimulation (*n* = 114, 27%)	7.08	5.44	1	30	0.51	0.47	0.00	2.96	27.19	6.37	1.60	36.00

*Note. N* = 370. Masturbation frequency = days of masturbation in one representative month in the past three months; Psychological distress = Global Severity Index of the Symptom-Checklist-27 (SCL-27 GSI); Sexual function = Total score of the Female Sexual Function Index (FSFI-d) (Berner et al., 2004).

#### Association between psychological distress and masturbation frequency

First, we examined whether higher psychological distress was associated with clitoral masturbation frequency or combined clitoral and vaginal masturbation frequency. We controlled for the significant covariates. Preliminarily analysis showed that relationship status and cohabitation were significantly associated with clitoral masturbation frequency. Post-hoc pairwise Wilcox-tests with Bonferroni correction showed significant differences in “single” and “close relationship” (*p* = .017). These results suggest a higher clitoral masturbation frequency for single people (*M*** **=** **10.81; *SD*** **=** **7.83) compared with people in monogamous relationships (*M*** **=** **8.14; *SD*** **=** **6.36). People not cohabiting with a partner (*M*** **=** **9.70; *SD*** **=** **5.74) also showed higher masturbation frequency compared to people cohabiting with a partner (*M*** **=** **7.56; *SD*** **=** **5.74). Since relationship status and cohabitation covary significantly (*χ*^2^ = 40.88, *p* < .001), we decided to only control for relationship status. Therefore, we dummy-coded relationship status and added the dummy-coded variables as covariates in the regression analysis. “Single” and “other relationship status” were included as dummy-coded variables, while “in a close relationship” was chosen as the reference category. Relationship status (Kruskal-Wallis chi-squared = 8.83, *p* = .012) showed significant associations with clitoral and vaginal masturbation frequency and was therefore included in the analyses. Again, pairwise Wilcox-tests with Bonferroni correction showed significant differences in “single” and “close relationship” (*p* = .012). Combined clitoral and vaginal masturbation frequency was higher in single participants (*M*** **=** **8.45; *SD*** **=** **5.20) than participants in a close relationship (*M*** **=** **6.43; *SD*** **=** **5.68). Thus, we dummy-coded “relationship status” in the same manner as before.

Regression analysis revealed that the SCL-27 total score significantly predicted clitoral masturbation frequency (*β* = 0.15, *p* = .015). Subscale analysis of the SCL-27 revealed that the subscales *dysthymic symptoms* (*β* = 0.21, *p* < .001) and *symptoms of mistrust* (*β* = 0.17, *p* = .007) significantly predicted clitoral masturbation frequency. Results are presented in Table 3. Results did not change if the previously excluded women were added to the sample again. To conclude, results indicate increased clitoral masturbation frequency with higher levels of psychological strain, independent of relationship status.

**Table 3.** Regression results with covariates for the association between psychological distress and clitoral masturbation frequency.

Variable	Regression model										Regression model (bootstrap)			
	*B*	*β*	*SE*	*t*	95% CI for *B*	*p*	*F*	df	*p*	Adj. *R*^2^	*B*	*SD*	95% CI for *B*	*p*
**Model global severity index**							5.36	3; 252	.001**	.049				
(Intercept)	6.72		0.78	8.58	[5.18, 8.26]	<.001***					6.74	0.73	[5.34, 8.18]	<.05*
Global severity index	2.60	0.15	1.06	2.40	[0.51, 4.69]	.015*					2.57	1.08	[0.44, 4.69]	<.05*
Single	2.63	0.17	0.97	2.71	[0.72, 4.54]	.007**					2.61	1.04	[0.67, 4.71]	<.05*
Other relationship status	3.41	0.11	1.93	1.77	[−0.39, 7.20]	.078					3.39	2.41	[−1.09, 8.39]	>.05
**Model depressive symptoms**							3.78	3; 252	.011*	.032				
(Intercept)	7.57		0.71	10.63	[6.17, 8.97]	<.001***					7.57	0.65	[6.31, 8.87]	<.05*
Depressive symptoms	0.91	0.07	0.75	1.20	[−0.58, 2.39]	.230					0.91	0.77	[−0.60, 2.44]	>.05
Single	2.65	0.17	0.98	2.71	[0.72, 4.58]	.007**					2.64	1.05	[0.66, 4.77]	<.05*
Other relationship status	3.45	0.11	1.96	1.77	[−0.40, 7.31]	.079					3.45	2.46	[−1.14, 8.50]	>.05
**Model dysthymic symptoms**							7.55	3; 252	<.001***	.072				
(Intercept)	6.46		0.71	9.14	[5.07, 7.86]	<.001***					6.46	0.68	[5.12, 7.78]	<.05*
Dysthymic symptoms	2.19	0.21	0.62	3.51	[0.96, 3.41]	<.001***					2.18	0.68	[0.86, 3.50]	<.05*
Single	240	0.15	0.96	2.49	[0.50, 4.29]	.013*					2.39	1.03	[0.45, 4.44]	<.05*
Other relationship status	3.04	0.10	1.91	1.60	[−0.71, 6.80]	.112					3.04	2.53	[−1.64, 8.25]	>.05
**Model vegetative symptoms**							3.35	3; 252	.027*	.027				
(Intercept)	7.95		0.68	11.76	[6.62, 9.28]	<.001***					7.95	0.60	[6.81, 9.15]	<.05*
Vegetative symptoms	0.40	0.03	0.89	0.46	[−1.34, 2.15]	.649					0.41	0.74	[−1.00, 1.90]	>.05
Single	2.70	0.17	0.98	2.74	[0.76, 4.63]	.007**					2.67	1.05	[0.70, 4.79]	<.05*
Other relationship status	3.74	0.12	1.95	1.92	[−0.09, 7.57]	.056					3.73	2.53	[−0.97, 8.97]	>.05
**Model agoraphobic symptoms**							3.54	3; 252	.015*	.029				
(Intercept)	7.89		0.60	13.05	[6.70, 9.08]	<.001***					7.90	0.57	[6.82, 9.03]	<.05*
Agoraphobic symptoms	0.85	0.05	0.99	0.87	[−1.09, 2.80]	.388					0.83	0.90	[−0.99, 2.57]	>.05
Single	2.77	0.18	0.99	2.81	[0.83, 4.72]	.005**					2.76	1.06	[0.72, 4.88]	<.05*
Other relationship status	3.84	0.12	1.94	1.98	[0.02, 7.66]	.049*					3.83	2.49	[−0.84, 8.88]	>.05
**Model sociophobic symptoms**							4.46	3; 252	.005**	.039				
(Intercept)	7.31		0.70	10.51	[5.94, 8.68]	<.001***					7.34	0.69	[6.02, 8.68]	<.05*
Sociophobic symptoms	1.25	0.11	0.68	1.84	[−0.08, 2.58]	.066					1.20	0.77	[−0.39, 2.67]	>.05
Single	2.58	0.16	0.98	2.65	[0.66, 4.51]	.009**					2.57	1.05	[0.60, 4.68]	<.05*
Other relationship status	3.89	0.13	1.93	2.01	[0.09, 7.69]	.045*					3.85	2.38	[−0.72, 8.68]	>.05
**Model symptoms of mistrust**							5.81	3; 252	<.001***	.054				
(Intercept)	7.05		0.66	10.65	[5.75, 8.36]	<.001***					7.06	0.59	[5.94, 8.20]	<.05*
Symptoms of mistrust	1.91	0.17	0.71	2.70	[0.52, 3.30]	.007**					1.90	0.74	[0.46, 3.37]	<.05*
Single	2.45	0.16	0.97	2.52	[0.54, 4.36]	.012*					2.44	1.05	[0.47, 4.53]	<.05*
Other relationship status	3.38	0.11	1.92	1.76	[−0.41, 7.16]	.080					3.37	2.42	[−1.16, 8.35]	>.05

*Note. N* = 256. 95% CI for B: confidence interval value for unstandardized beta values. Signif. codes: 0 “***” 0.001 “**” 0.01 “*” 0.05 “.” 0.1.

### Qualitative results

Next, we explored in what ways the relationship between psychological distress and masturbation frequency was explained by expressed feelings about masturbation in women’s self-reports. We created three higher-level categories labeled *reasons for masturbation* (*n*** **=** **60; 16%), *positive effects of masturbation* (*n*** **=** **312; 84%), and *negative effects of masturbation* (*n*** **=** **63; 17%) during QCA. They contain a total of four concept-driven (CD) and three data-driven (DD) categories, which are presented below. Three other categories generated by QCA, namely *positive bodily sensations* (*n*** **=** **118; 31%), *feeling indifferent about masturbation* (*n*** **=** **6, 2%), and *sexual self-esteem* (*n*** **=** **115, 31%), were not deemed relevant to the current research question.

#### Reasons for masturbation

The first major theme includes the subcategories *using masturbation as a coping mechanism* (*n*** **=** **44; 12%; CD) and *using masturbation for well-being and self-care* (*n*** **=** **20; 5%; DD). Several women expressed that they deliberately used masturbation as a means of stress relief and as a strategy for dealing with stressful situations, difficulty falling asleep, and pain. One woman wrote: “For me, it is also a valve to relieve stress. I notice that I masturbate more often during exam periods, for example” (No. 375; aged 23; bisexual). Others mentioned that masturbation served as a distraction, with one participant saying: “It [masturbation] distracts me and relieves me from stress.” (No. 144; aged 19; heterosexual) while someone else said: “It [masturbation] brings me back to the here and now and makes me forget everything else for a moment.” (No. 292; aged 28; heterosexual). Few women (*n* = 7; 2%) mentioned using masturbation for pain relief or to help them fall asleep: “It [masturbation] helps me fall asleep.” (No. 353; aged 30; heterosexual).

Furthermore, participants highlighted the positive impact of masturbation on their mental health and general well-being, often referring to it as “me-time” (e.g., No. 357; aged 26; heterosexual), “self-care” (e.g., No. 70; aged 31; heterosexual), or a way of “taking care of myself (doing something good for myself)” (e.g., No. 74; aged 22; bisexual). One woman wrote: “It contributes to psychological and physiological health.” (No. 389; aged 51; heterosexual).

#### Positive effects of masturbation

The second major theme consists of two subcategories: *evoking joyful and uplifting emotions* (*n*** **=** **203; 55%; CD) and *evoking calming and relaxing feelings* (*n*** **=** **238; 64%; CD). Most participants reported that masturbation evoked joyful and uplifting emotions, such as “happiness” (e.g., No. 79; aged 21; bisexual), “excitement” (e.g., No. 63; aged 20; bisexual), or “joy” (e.g., No. 70; aged 31; heterosexual). Additionally, a lot of women associated calming and relaxing feelings with masturbation. They mentioned feeling “calm” (e.g., No. 475; aged 25; heterosexual), “relaxed” (e.g., No. 63; aged 20; bisexual), “content” (e.g., No. 436; aged 28; heterosexual), and “comfortable” (e.g., No. 316; aged 27; heterosexual). They also mentioned a general sense of well-being and highlighted that masturbation helped them to switch-off and let go.

#### Negative effects of masturbation

The last major theme includes three subcategories: *negative emotions* (*n*** **=** **48; 13%; CB), *social stigma surrounding masturbation* (*n*** **=** **20; 5%; DB), and *preference for partner stimulation* (*n*** **=** **5; 1%; DB). Women described experiencing negative emotions related to masturbation, such as feeling weird, disgusted, lonely, or ashamed at present (*n*** **=** **32, 9%) or during their younger years (*n*** **=** **4, 1%). These feelings were attributed to masturbation itself, sexual fantasies, or consumption of pornography. For instance, one woman said: “I feel a little bit ashamed. I wouldn’t necessarily want to talk to anyone about it.” (e.g., No. 511; aged 24; demisexual). Some women also expressed frustration or anger when they were unable to achieve orgasm due to low libido or lack of knowledge about appropriate stimulation (*n*** **=** **12, 3%). Few women perceived masturbation as a taboo subject in society, particularly for women. Someone stated: “Female masturbation is often still a taboo subject, you can’t really talk about it openly.” (e.g., No. 121; aged 19; heterosexual). Lastly, a small number of women expressed a strong preference for partner stimulation or partnered sexual activities over masturbation, stating that it was not as satisfying as being with a partner. One participant mentioned, “It’s [masturbation] good, but not as good as with a partner” (e.g., No. 270; aged 21; heterosexual).

### Mixed-method results

Mixed-method analysis was used to determine to what extent the quantitative data might be explained by the qualitative data. Qualitative categories were dichotomized and then used for the statistical analyses with the quantitative data. Group differences were calculated using one-sided Welch two sample *t*-tests using a significance level of *p* < .001 to correct for multiple testing, while a significance level of *p* < .05 was used for Chi-Squared tests.

#### Group differences in psychological distress for women reporting and not reporting reasons for masturbation

One-sided Welch two sample *t*-tests revealed that women who indicated a reason for masturbation did not differ in their psychological stress (SCL-27 GSI) from women who did not indicate a reason for masturbation (*t*_(368)_ = 0.12, *p* = .549, Hedges’ *g* = 0.16). Likewise, women who explicitly indicated to use of masturbation as a coping strategy (*t*_(368)_ = 1.12, *p* = .132, Hedges’ *g* = 0.02) or for well-being and self-care (*t*_(16)_ = 1.73, *p* = .051, Hedges’ *g* = 0.46) did not experience significantly higher psychological distress than women who did not receive those codes. Analysis using the subscales of the SCL-27 instead of the GSI did not produce any significant effects either.

#### Group differences of psychological distress in women reporting and not reporting negative effects of masturbation

Analysis revealed that women who reported negative effects of masturbation did not differ in their psychological distress (GSI) from women who did not indicate negative effects (*t*_(91)_ = 1.47, *p* = .073, Hedges’ *g* = 0.19). Women who reported negative feelings from masturbation (*t*_(79)_ = 0.15, *p* = .442, Hedges’ *g* = 0.02), who reported social stigma for masturbation (*t*_(20)_ = 2.11, *p* = .024, Hedges’ *g* = 0.61) and women who reported to prefer partner stimulation (*t*_(4)_ = 0.31, *p* = .386, Hedges’ *g* = 0.14) did not significantly differ in their psychological distress from women not indicating those categories. Subscale analysis of SCL-27 did not alter results.

#### Association of positive effects of masturbation with mode of stimulation

Chi-Squared tests showed that whether women reported that masturbation evokes joyful and uplifting emotion or not was not dependent on the mode of stimulation (*χ*^2^ = 2.23, *p* = .135). Neither were reports of calming and relaxing emotions evoked by masturbation (*χ*^2^ = 0.58, *p* = .448).

## Discussion

In the present study, we investigated the relationship between psychological distress and masturbation behavior in women, and we explored the role of clitoral and vaginal stimulation using a concurrent mixed-method design. A total of 370 women participated in the study, providing detailed information about their masturbation behavior within the past three months and responding to an open-ended question. Our findings align with previous results, indicating that (clitoral) masturbation serves as a reliable behavioral coping mechanism for psychological distress in women (e.g., Burri & Carvalheira, 2019; Meiller & Hargons, 2019). The results of our study support the perspective that masturbation is predominantly beneficial to mental health and emphasize the importance of considering the mode of stimulation for future research.

Regarding our first hypothesis, our findings back the notion that psychological distress is associated with higher masturbation frequency using clitoral stimulation. This aligns with previous studies indicating that clitoral stimulation is often considered the fastest, easiest, and most reliable means of sexual arousal (Jannini et al., 2012; King et al., 2011; Sayin, 2017). Consequently, clitoral stimulation is more likely to be utilized for stress relief, which may explain the observed association between psychological distress and clitoral stimulation. This idea is further supported by a study that found women who exclusively use clitoral stimulation during masturbation are more inclined to report stress relief and relaxation as their primary motivations for masturbation (Rowland et al., 2020). This can additionally be interpreted based on the orgasm gap between men and women (Frederick et al., 2018) and gender inequality regarding sexual pleasure in sexual interactions (Laan et al., 2021) that might lead women to choose clitoral masturbation as an uncomplicated way to achieve relaxation during stressful times. These findings underscore the crucial role of the clitoris in female sexuality and stress reduction.

The frequency of combined clitoral and vaginal stimulation was not related to psychological distress in our study, even though levels of psychological distress did not differ between women using clitoral stimulation alone and women using combined clitoral and vaginal stimulation. Similar to previous research, these findings indicate that masturbation incorporating vaginal stimulation is a less common form of stimulation in women (Burri & Carvalheira, 2019; de Lima et al., 2022; Rowland et al., 2020). We speculate that the inclusion of vaginal stimulation in masturbation is a sign of sexual exploration and discovery rather than one of dealing with psychological distress.

In the current study, we found that the association between psychological distress and clitoral masturbation frequency was specific to certain aspects of psychological distress, particularly the general level of psychological distress and the subscales of dysthymic symptoms and symptoms of mistrust. Previous research on the relationship between psychological distress and sexual behavior has predominantly focused on symptoms of anxiety and depression, with findings suggesting facilitative, inhibitory, or neutral associations (Kane et al., 2019; Soler et al., 2021). However, there is limited research investigating the specific differences in types of psychological distress and their relation to sexual behavior.

Generally, symptoms of depression have been found to interfere with sexual arousal (Graham et al., 2004). However, two studies propose that women with depressive symptoms might turn to masturbation as a means of enhancing mood and experiencing pleasure they may not experience otherwise, thus serving as a form of mood repair (Csako et al., 2022; Frohlich & Meston, 2002). In our study, dysthymic symptoms seemed to be relevant for masturbation frequency, while depressive symptoms were not. Dysthymic symptoms are characterized by less severe but chronic depressive symptoms that have a significant impact on daily functioning (Sekhon & Gupta, 2022), and they are often overlooked by individuals and clinicians (Schramm et al., 2020). It is possible that a higher frequency of masturbation serves as a coping mechanism for individuals in non-clinical samples to deal with dysthymic symptoms. This indicates that women may successfully utilize pleasant-seeking behaviors like masturbation to regulate negative mood, dissatisfaction, and anhedonia, as indicated by our qualitative results.

In line with this research, our results suggest that the association between psychological distress and sexual behavior is complex and shows variability depending on the specific type of psychological distress. Further research is needed to establish whether there are clear differences in how symptoms of anxiety and depression are related to sexual behavior.

Qualitative results provide valuable insights into the reasons behind the association between psychological distress and increased masturbation frequency. The majority of women in our study reported positive effects of masturbation, such as feelings of joy, happiness, relaxation, and calmness. Negative emotions following masturbation were reported by only a few women, indicating a shift away from predominantly negative to predominantly positive perceptions of female masturbation in modern Western societies. According to the women in our study, masturbation serves as a strategy to induce positive emotions and promote relaxation when experiencing stress. These findings are consistent with previous research that has highlighted the role of masturbation as a coping strategy for (psychological) stress and as a means of relaxation (Csako et al., 2022; de Lima et al., 2022). In our study, some women also reported that masturbation helped them deal with pain and improve their ability to fall asleep. These findings align with previous research on the physiological effects of masturbation on pain management and sleep quality (Gianotten, 2021; King et al., 2021; Lastella et al., 2019; Meiller & Hargons, 2019).

As mentioned above, previous research was concerned about the possibility that clitoral masturbation might be a sign of missing adaptive coping strategies in women (Brody & Costa, 2008). In our study, some women reported using masturbation as a means of distraction, which can be interpreted as a strategy to avoid experiencing negative feelings. When determining whether masturbation serves as a beneficial or detrimental coping strategy, it is crucial to carefully consider the underlying motivation for engaging in masturbation. If masturbation is intentionally employed as a proactive strategy for pain relief, relaxation, or improving sleep, the short-term positive effects of masturbation are expected to be beneficial to mental health. In this context, we would consider masturbation as an effective behavioral coping strategy. However, it is important to note that individual motivations and circumstances may vary, and further research is needed to fully understand the complexities of the relationship between masturbation and coping strategies in different individuals and contexts.

The mixed-method analysis used in this study aimed to provide a more comprehensive understanding of the relationship between masturbation, coping strategies, and psychological distress. However, the findings did not align with the initial expectation that women using masturbation as a coping strategy or for well-being and self-care would have higher levels of psychological distress. One possible explanation for this unexpected result is that only a relatively small percentage of women (16%) indicated using masturbation as a coping strategy or for well-being and self-care. This lower percentage compared to previous studies may have influenced the overall findings (Burri & Carvalheira, 2019; Carvalheira & Leal, 2013; Csako et al., 2022; de Lima et al., 2022). It is possible that a more direct and specific question regarding the reasons for masturbation or the use of masturbation as a coping strategy could have yielded different responses and potentially revealed stronger associations with psychological distress. Furthermore, our analysis indicated that negative feelings resulting from masturbation were not associated with higher levels of psychological distress, supporting the notion that masturbation predominantly has beneficial effects. Regarding the comparison between clitoral stimulation and combined clitoral and vaginal stimulation, the analysis did not reveal significant differences in reported effects of masturbation between the two groups. One possible explanation for this is that clitoral stimulation was present in both groups, making it challenging to differentiate the effects of clitoral stimulation alone *vs.* combined clitoral and vaginal stimulation. Another explanation is that clitoral stimulation itself may be as relaxing as vaginal stimulation, and the reported effects of masturbation may not differ significantly between the two modes of stimulation. Some scholars argue that there are no differences between clitoral and vaginal stimulation since stimulation of the vagina simultaneously leads to activation of the clitoris, proposing that the clitoris and vagina function as a unit (Buisson et al., 2010; Levin, 2020).

In all, our findings show that clitoral stimulation is associated with psychological distress, while combined clitoral and vaginal stimulation is not. Additionally, no differences were found between the effects of masturbation and the mode of stimulation. Results motivate to further explore a bidirectional model in which psychological distress motivates masturbation behavior, while the resulting positive effects of masturbation increase the likelihood of future masturbation behavior for dealing with psychological distress (Fleischman, 2016).

### Limitations and implications for future research

To our knowledge, this study is the first to investigate the use of masturbation as a self-care strategy and as a strategy for stress reduction while considering the different psychophysiological effects of clitoral and combined clitoral and vaginal stimulation in this context. One addition of our study to previous work is the mixed-method study design, which allowed us to enrich our understanding of the quantitative findings through triangulation with qualitative data in a large sample of women. This provides greater validity of data and higher confidence in the interpretation of the data compared to previous studies.

The current study has several limitations. First, the cross-sectional design limited our ability to determine how using masturbation as a coping strategy and for well-being might be related to psychological distress over time. We do not know whether these effects are short-term or also translate into long-term effects. A design where current mood and well-being are assessed before and after masturbation would be most advantageous. This could establish how mood before and after masturbation translate into general well-being and thus help understand the underlying processes of the association between psychological distress and masturbation. Furthermore, it should be established whether it is the experience of orgasm or masturbation behavior that is the reason for positive effects on psychological distress. However, these results remain qualitative in nature and future research will need to determine to what extent masturbation might function as a beneficial form of coping with psychological distress, sleeping problems, and pain. Additionally, future studies should not only account for the site of stimulation but also the method used (hand, sex toy, etc.) since vibrators and air pulse stimulation devices are associated with more reliable and quicker orgasms (Herbenick et al., 2009).

There are also limitations regarding the measures used in this study. While psychological distress was assessed during the last week, masturbation frequency was assessed by monthly masturbation days. We strongly urge researchers to use the same time span for psychological distress and masturbation frequency for further studies.

Additionally, this study relied on retrospect self-report, which is prone to recall bias. The used questionnaire for psychological distress in this study made it difficult to accurately differentiate between symptoms of anxiety and depression. Future studies should put effort into distinguishing symptom-specific associations with masturbation, which would additionally clarify implications for new screening instruments for psychological distress. We recommend that sexual activity should be assessed separately for individual and partnered sexual behaviors in women and it should be considered that there is variability in how type of psychological stress is related to sexual behavior in women.

Regarding the methods used in the study, we note additional limitations. Although we excluded women using additional stimulation during masturbation, we did not control for additional partnered sexual activities. This was due to existing literature suggesting that masturbation behavior in women is not dependent on partnered sexual activities. Furthermore, the fact that almost no women indicated to use only vaginal stimulation during masturbation limited our ability to differentiate between the effects of clitoral and vaginal stimulation. Further studies are needed to determine if vaginal stimulation alone might result in different effects than clitoral stimulation. Pertaining to the qualitative analysis, to limit subjectivity during coding, we based our categories on previously published evidence, and additional inductive categories were discussed amongst the team. Additionally, we double-coded the qualitative data to limit subjective interpretation. Our qualitative question was relatively vague and open to interpretation. This enhanced the variety in results but also limited our ability to deduce the actual percentage of women who use masturbation as a coping strategy or for well-being and self-care.

Another limitation concerns the nature of the convenience sample, which differs from the general population of women regarding age, education, and level of psychological distress. Collecting data online could have limited the participation of women, namely older women, and women who did not have access to social media.

Even though the anonymity of the survey should have decreased the effect of social desirability, this may not have been prevented entirely. Sex-positive attitudes may have been overrepresented in this sample because women with less interest in sexual activities may have refrained from participation in the study. It can be assumed that this sample represented open-minded, modern women, while more conservative or reluctant subjects refrained from participation.

To better understand masturbation behavior, the hypothesis that incorporating vaginal stimulation when masturbating may be a sign of exploration should be further addressed.

### Conclusion

Our study is the first to investigate masturbation as a coping strategy when dealing with psychological distress with a mixed-methods approach. While masturbation has been marginalized for many decades by medical, societal, and academic discourses (Coleman, 2003; Ford et al., 2021), this study supports the notion of mostly beneficial effects of masturbation.

Our findings have implications for researchers, society, and clinical practitioners. For researchers, it is important to consider the mode of stimulation and type of sexual activity when assessing the link between psychological distress and sexual behavior and use mixed-method study designs. For society, this study highlights the great potential of using masturbation as a coping strategy and/or means of self-care. Interestingly, some companies are now even considering giving employees paid time for sexual activities in Sweden (Bilefsky & Anderson, 2017), which represents the growing trend of recognizing the link between stress and sexual pleasure. Our research aligns with the potential benefit of this, especially for women who experience much stress. However, many women are not yet aware of the positive implications for their health and general well-being (Gianotten et al., 2021). This could be changed by psychoeducation and specific health promotion programs. With psychological distress on the rise (e.g., Schafer et al., 2022; Twenge et al., 2018), women across cultures could benefit from the knowledge as part of health literacy, since masturbation provides an easily accessible, safe, and effective way for self-care and relaxation. The education system could fully embrace sex- and pleasure-positive sex education encouraging girls to explore their bodies. Lastly, clinicians can benefit from the results of this study by incorporating masturbation into their treatments. Our results provide initial indications of the disorders for which this might be useful. Further studies should provide additional knowledge regarding the differential diagnosis so that in the future there will be a clearer idea for whom, when, and for which problems masturbation may be recommended. Our results may encourage clinicians to implement psychoeducation about masturbation and to consider masturbation as a way of maintaining self-care as well as a resource for dealing with psychological distress and pain in sex-positive patients. Clinicians in sexual health, mindfulness therapy, pain management training, stress-specific training, or couple therapy could benefit by including sexuality in their psychological treatment protocol, following a more holistic model of psychological health.

## Data Availability

Data is available on OSF: https://osf.io/cgys2/?view_only=a740e72afb794f148b4a1db8489a6112.

## Code availability statement

Code is available on OSF: https://osf.io/cgys2/?view_only=a740e72afb794f148b4a1db8489a6112.
